# Saliency at first sight: instant identity referential advantage toward a newly met partner

**DOI:** 10.1186/s41235-019-0186-z

**Published:** 2019-11-04

**Authors:** Miao Cheng, Chia-huei Tseng

**Affiliations:** 1 NTT Communication Science Laboratories, NTT Corporation, Atsugi, Japan; 2 Department of Psychology, University of Hong Kong, Hong Kong SAR, China; 3 Research Institute of Electrical Communication, Tohoku University, Sendai, Japan

**Keywords:** Partner-advantage, Self-advantage, Self-bias, Identity referential advantage, Familiarity

## Abstract

Neutral information enjoys beneficial processing when it is associated with self and significant others, but less is known about how the identity referential advantage is constructed in the initial stages of a relationship. We offer a novel solution by asking if a newly met stranger could provide a processing advantage in a shape-identity matching task where shapes were associated with the names of different identities. Each participant was paired with a newly met partner in a joint shape-identity matching task in which three shapes were associated with the names of the participant or his/her best friend, the partner, and a stranger, respectively. The participants judged whether or not the shape and name correctly matched.

Intriguingly, the trials related to a newly met partner exhibited instant referential saliency, which was more accurate and faster than that related to the stranger’s name (baseline) when the partner was physically present (experiments 1, 2, 4, 5), but not when the partner was absent (experiment 3). Self-advantage, however, was robust and lasting. The precursor of physical presence when forming referential saliency toward a stranger and its distinct temporal dynamics imply a novel referential benefit unendowed with familiarity, which is qualitatively different from the well-documented self/friend-advantage effect.

**Electronic supplementary material:**

The online version of this article (10.1186/s41235-019-0186-z) contains supplementary material, which is available to authorized users.

## Significance

During a social interaction, the most important first step is to identify the participants and their relationship to us (e.g. superiors, friends, subordinates). The ability to process people’s identities helps us to interact appropriately in all social contexts. Extensive research has shown that we prioritize processing for identities that are important and familiar (e.g. mother, friend, and self). Neural objects associated with self and significant others are processed faster and more accurately than objects associated with strangers; we also perceive objects and events associated with important identities more favorably, remember them better, and pay more attention to them. However, we meet new people all the time, and it is unknown whether similar modulations occur at the initial stage of a relationship or how identity-advantage develops over time. Most scientific studies focus on the advantage effect in relation to identities with high social significance and high familiarity and so have been unable to inform us about this missing information as regards strangers. We designed a critical study to successfully observe the identity-advantage toward a newly met partner with minimal intercourse. We discovered a distinct time-course based on priority processing for self and significant others, and that the physical appearance of the partner is critical. This is the first study exploring the minimal requirement for provoking an identity referential advantage in the early stages of a social relationship. It is likely to be served by a dissociable pathway independent from the pathway that processes long-term personal significance. Our research provides a new theoretical perspective of identity referential processing and poses a well-defined working hypothesis for future studies to investigate the dissociable mechanisms that underlie slow and fast pathways for other-referential advantage.

## Introduction

The processing priority of a stimulus is modulated when attached to identities (e.g. self, friend, or stranger). The most extensively studied case in identity referential processing, namely self-advantage (also known as self-bias), refers to the facilitation of performance toward stimuli related to one’s “self”, such as one’s own face (Ma & Han, 2010; Sui & Humphreys, 2013; Tong & Nakayama, 1999) and name (Harris, Pashler, & Coburn, 2004; Moray, 1959), as opposed to stimuli related to other identities. This benefit also extends to neutral stimuli newly associated with one’s self, such as geometric shapes (Sui, He, & Humphreys, 2012), movements (Frings & Wentura, 2014), concrete objects (Cunningham, Brebner, Quinn, & Turk, 2014), and abstract concepts (Schäfer, Wentura, & Frings, 2015). It has been proposed that the sense of self serves as glue, interlinking various functions such as perception, attention, memory, and decision making (Humphreys & Sui, 2016; Northoff, 2016; Sui & Humphreys, 2017).

A similar advantage also applies to significant others such as friends and mothers, although to a lesser extent. Better performance was observed in a face identity classification task when friends’ rather than strangers’ faces were present (Sui & Humphreys, 2013) and in a shape-identity matching task when shapes were associated with a friend or mother than with a stranger (Sui et al., 2012) or a neutral object (Schäfer, Frings, & Wentura, 2016). Zhang and colleagues (Zhang, Zhu, & Wu, 2014) asked participants to judge a total of 144 traits of self, a close friend, and a celebrity, followed by a surprising memory recognition task on trait adjectives. They found that if the trait appeared in a question about a friend, it was better recognized than celebrity-related traits.

Whether or not there are distinct mechanisms underlying self-advantage and other-advantage is currently unknown. Research has shown that other-advantage is easily weakened, and even eliminated, by low-rewarded association (Sui et al., 2012; Sui & Humphreys, 2015), degradation of stimulus probability (Sui, Sun, Peng, & Humphreys, 2014), and luminance contrast (Sui et al., 2012). Lack of robustness indicates that other-advantage may have the plasticity needed to allow it to be easily built or shaped. In contrast, self continues to enjoy absolute priority of processing, which is difficult to attenuate by perceptual degradation. The difference in robustness was taken to suggest that the mechanisms underlying the performance advantage toward self and significant others are essentially different.

However, because the above-reported other-advantage occurs in identities with strong familiarity and social significance, such as mother and best friend, it is still unknown how identity referential advantage is formed and whether it is involved in the early stage of relationships. We investigate this topic by testing whether or not identity-advantage can be created toward a newly met stranger. We adopted the shape-name association task described in Sui et al. (2012), and introduced a new identity: a newly met stranger to co-act with participants as partners in a joint task (Table 1). The task required participants to associate three geometric shapes (square, circle, and triangle) with three names. The names were own name or best friend’s name, a newly met partner’s name and a unisex neutral stranger’s name (君明, i.e. Jun Ming in Mandarin). Participants were presented with a pair consisting of a name and a shape and required to judge whether or not they matched. The performance in these three identity categories was analyzed as an index of identity priority processing.

**Table 1:**
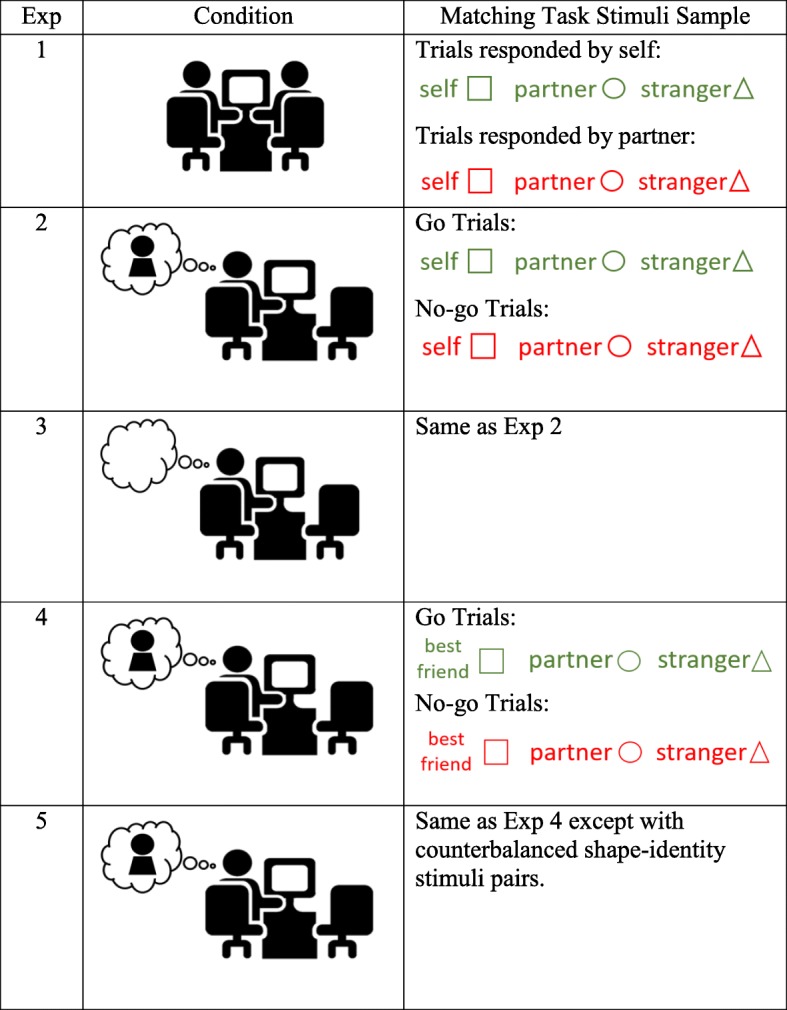
Experimental conditions and matching task. In experiment 1 (the joint condition), two participants performed the task on separate keyboards. Each participant responded only to their assigned color (red or green) in the task (go trials). In this example, their names and a neutral name were assigned to correspond to three shapes (square, triangle, and circle), which participants had to memorize for the name-shape matching task. In experiments 2, 3, 4, and 5 (individual condition), the other participant waited outside the room. Similarly, participants responded only to their assigned color (go trials) but not the other color (no-go trials). In experiments 2, 4, and 5, the participants briefly met the partners without any verbal communication, while in experiment 3, the participants never met the partners. Experiment 5 was a control experiment to replicate experiment 4 with counterbalanced stimuli

In experiment 1, we investigated whether familiarity is a prerequisite for identity-advantage by examining whether we could promote identity processing toward a joint action partner with minimum familiarity with participants. We then removed the components comprising “partner” one by one. In experiment 2, the participants met a new partner briefly without actually performing the joint task together. In experiment 3, participants were introduced to their partner by name only without any physical presence. Experiment 4 was to estimate whether the process advantage associated with the newly met partner was comparable to friend-advantage. Experiment 5 was a control experiment. Together, our results suggested that an identity referential advantage could be established toward a stranger without prior familiarity or a close relationship to self.

### Experiment 1

#### Purpose

The aim of this experiment was to investigate whether or not an identity referential advantage can be created towards a co-acting partner in a joint action context.

#### Participants

The sample size was determined based on previous studies (e.g. Sui et al., 2012). A significant self-advantage and friend-advantage was reported as a result of analysis of variance (ANOVA) tests of sensitivity (*d* prime (*d*’)) and response time (RT) conducted on 18 participants with effect sizes (*η*^2^) of 0.41 and 0.67 respectively. We anticipate a smaller effect size here because we replaced friends with a newly met partner. Therefore, with an estimated *η*^2^ of 0.2, we expected an effect size *f* of 0.50. Then a power analysis that we conducted using G*Power (Faul, Erdfelder, Lang, & Buchner, 2007) yielded a sample size of 22 needed for the *F* test (ANOVA, repeated measures, within factors) to for power of 0.8 with the alpha value at 0.05. In reality, 22 university students participated in this study (10 men and 12 women), all with normal or corrected-to-normal vision.

Each time, we studied two participants who were strangers who had never met prior to the experiment. Due to participant no-show, two participants were paired with a confederate, and we verified that they had not met the confederate before.

#### Stimuli

**Fig. 1:**
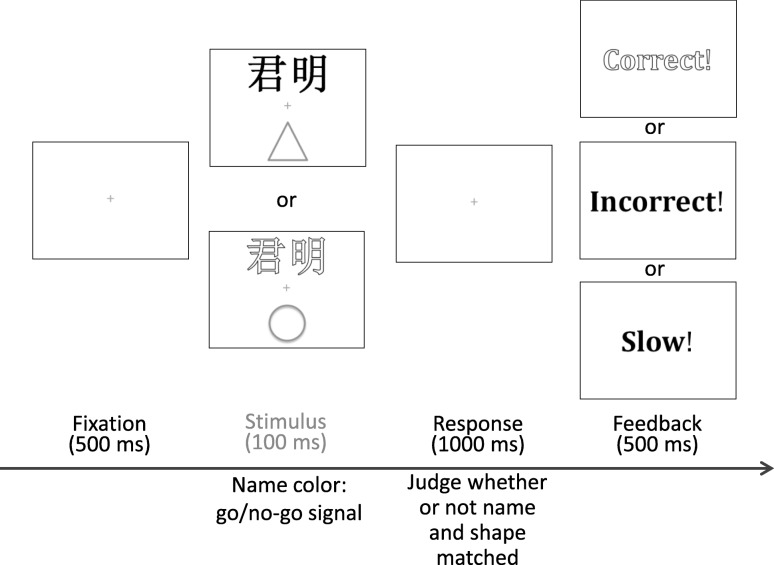
Experimental procedure. The stimuli in the experiment were presented against a black background. Stimuli with an open font represent words colored in red; those with a solid font represent words colored in green

The task required the participants to associate three geometric shapes (square, circle, and triangle) with three names. The names were those of two participants and a neutral name. To control the length of the names, they all consisted of two Chinese characters. The last two characters (first name) were used if a participant’s name had three characters. A unisex name (君明) was used as the neutral name. The participants confirmed that they had not known anyone with either of the other two names before the experiment. The stimuli were shown against a black background. One name (visual angle of 4.3° × 2.2°) was presented above a central gray fixation cross (visual angle of 0.5° × 0.5°), and one shape (visual angle of 3.4° × 3.4°) was presented below the fixation cross (Fig. 1). The distance between the names or shapes and the fixation was a visual angle of 7.2°. The experiment was conducted with E-prime software (Version 2.0) on a 24-in. Dell monitor (E248WFPb, 1440 × 900, 60 Hz).

#### Procedure

The participants were briefly introduced to each other upon arrival (“this is your partner, XXX (partner’s name)”) and were informed that they were later going to perform a task together. The two participants were separated before any further opportunities for verbal communication, and took turns to receive instruction and practice trials in the experimental room while the other waited outside. The participants were instructed to remember matching rules for three names and three shapes: self was associated with a square, partner with a circle, and triangle with a neutral stranger who never showed up (君明). The rules were identical for all the participants. Each participant undertook 30 practice trials alone before performing 450 trials in the formal experiment (3 blocks, 150 trials each) sitting beside their partner in front of one monitor with separate response keyboards (see Table 1).

Figure 1 summarizes the experimental procedure. All the trials started with a fixation cross presented for 500 ms, followed by the name-shape pairing stimulus for 100 ms. The name was written in red or green. The color served as a go/no-go signal. Within each pair, one participant responded only to red names and the other only to green names. The participants needed to indicate whether the name and the shape matched or not as accurately and quickly as possible with two keys (“n” or “v” for one participant, “1” or “3” for the other). After the response had been received, visual feedback (“Correct” or “Incorrect”) was displayed for 500 ms. If the participants did not respond within 1000 ms, the program displayed feedback consisting of the word “Slow” for 500 ms, and the next trial began. Slow trials were considered inaccurate responses during data analysis. When the participants responded in the no-go trial (partner’s trial), they also received the feedback “Incorrect”. If both participants responded to one trial, only the faster response was recorded and feedback was provided accordingly. The participants were instructed to maintain overall accuracy while responding as quickly as possible. There was no special reward for any specific identity category, thus ensuring that the participants would not strategically favor any particular identity to maximize the reward. The entire experiment lasted about 30 min.

#### Results

Results obtained from all 22 participants were included in the data analysis. Data from two participants in one session were processed individually (i.e. not combined for pair analysis). Responses faster than 200 ms were excluded, eliminating a total of 3.6% of the trials. Only accurate trials were included in the RT analysis and 8.4% of trials in which there were erroneous responses were excluded.

To investigate identity-related advantage, we compared the *d’* and RT in trials relating to different identities using ANOVA. Accuracy data were also analyzed for all experiments and for reference are shown in supplementary figures in the Additional files. Individual *d*’ plots by experiment are also available in the Additional files. We used *d*’ (the difference between the Z scores of the hit rate and false alarm rate) as an index of performance in previous identity referential advantage research (Stenzel & Liepelt, 2016; Sui et al., 2012) and in the current study, because it takes both hit rate and false alarm rate into consideration and better reflects the sensitivity of response to different identity-shape pairs. We considered there to be an advantage effect if it was observed in either *d’* or RT.

**Fig. 2:**
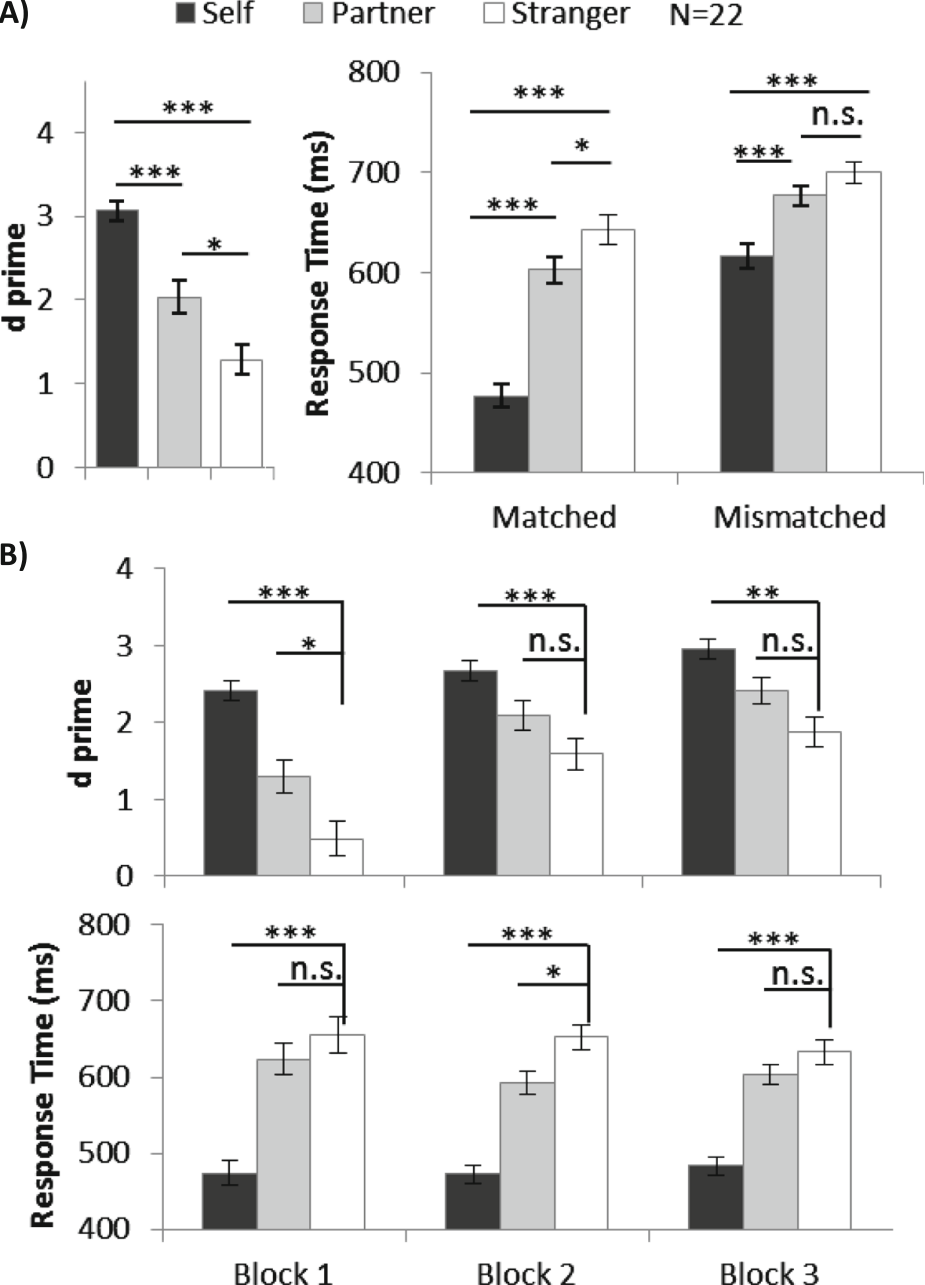
**a** Mean and SE of *d* prime (*d’*) and response time (RT) for different shape categories in experiment 1. **b** Mean and SE of *d’* and RT (matched trials) for different shape categories for each block in experiment 1 (*p < .05, **p < .01, ***p < .001). n.s., not significant

For the sensitivity analysis, we employed a signal detection approach and calculated *d’* from both matched and mismatched trials. Extreme hit rate and false alarm rate was adjusted using the log-linear correction method (Hautus, 1995). The *d’* values for individual participants are shown in Additional file 6: Figure S6 and the average results are summarized in Fig. 2a. Average accuracy and accuracy by block are shown for reference in Additional file 1: Figure S1. We employed one-way ANOVA with a within-subjects factor shape category (self-associated, partner-associated, and stranger-associated), and we found a significant main effect, *F* (2, 42) = 39.09, *p* < .001, *η*^2^ = 0.65. A post-hoc test with Bonferroni adjustment showed a higher *d’* in self-related trials (*d’* = 3.06) than in stranger-related trials (*d’* = 1.29, *p* < .001). Furthermore, the participants performed more accurately in partner-related trials (*d’* = 2.03) than in stranger-related trials (d’ = 1.29, *p* = .02). These results indicate that, when compared with stranger, both self and partner were processed more accurately under the joint action condition (Fig. 2a).

The response time results are summarized in Fig. 2a. Two-way ANOVA with within-subject factors of shape category and matching judgement (matched and mismatched) revealed a significant interaction effect between shape category and matching judgement, *F* (2, 42) = 25.26, *p* < .001, *η*^2^ = 0.55. Therefore, we conducted follow-up ANOVA separately on matched and mismatched trials. In the matched trials, there was a significant main effect of shape category, *F* (2, 42) = 113.03, *p* < .001, *η*^2^ = 0.84. Post hoc tests showed self-related trials (476.97 ms) and partner-related trials (602.57 ms) exhibited a significantly quicker response than stranger-related trials (642.63 ms, *p* < .001 and *p* = .04, respectively). Self-advantage was observed in mismatched trials, (616.72 ms vs 699.75 ms, *p* < .001), but partner-advantage was not (676.85 ms vs 699.75 ms, *p* = .19).

To further investigate the unexpected partner-advantage, we examined its temporal dynamics block by block: we would expect a gradual improvement across blocks if it is a process similar to familiarization. However, if it is a process similar to first-impression endowment, we would expect this advantage to appear very early in the experiment, i.e. block 1. Therefore, we compared the performance of three identities block by block by planned pairwise comparison and Bonferroni adjustments. The *d*’ value was calculated and used for the sensitivity analysis, and the RTs from matched trials were used for speed analysis. Effect size was calculated using Cohen’s *d*_rm_ (Lakens, 2013) for multiple comparison tests in repeated measures ANOVA:

$$ Cohe{n}^{\prime }s\ {d}_{rm}=\frac{Mean_1-{Mean}_2}{\sqrt{\mathrm{S}{{\mathrm{D}}_1}^2+\mathrm{S}{{\mathrm{D}}_2}^2-2\times \mathrm{r}\times \mathrm{S}{\mathrm{D}}_1\times \mathrm{S}{\mathrm{D}}_2}}\times \sqrt{2\left(1-r\right)} $$

in which *r* is the correlation coefficient of two groups.

The results are summarized in Table 2 and Fig. 2b. We observed a robust self-advantage through 3 blocks: participants responded significantly more accurately and faster with a self-related stimulus than with a stranger-related stimulus. Partner-related associations were significantly stronger than stranger-related associations in block 1 (in *d’*) and block 2 (in RT), but not in block 3.

**Table 2:** Planned pairwise test results (*p* value and effect size of Cohen’s *d*_rm_) with post-hoc Bonferroni adjustments on the *d* prime (*d*’) and response time (RT) of matched trials of averaged data across blocks and block-by-block data, and in all experiments

Exp	*p* value/ Cohen’s *d*_rm_	All blocks		Block 1		Block 2		Block 3	
		Self/friend- bias	Partner-bias	Self/friend-bias	Partner-bias	Self/friend-bias	Partner-bias	Self/friend-bias	Partner-bias
1	Dprime	**<.001/2.53**	**.02/0.87**	**<.001/2.23**	**.02/0.78**	**<.001/1.32**	.34/0.54	**.001/1.39**	.09/0.62
	RT	**<.001/2.90**	**.04/0.56**	**<.001/1.70**	.39/0.29	**<.001/2.60**	**.02/0.81**	**<.001/2.10**	.34/0.42
2	Dprime	**<.001/1.49**	**.006/0.63**	**<.001/1.57**	**.033/0.53**	**<.001/1.43**	**.022/0.64**	**.002/0.89**	.11/0.46
	RT	**<.001/2.56**	**.01/0.61**	**<.001/2.34**	**.04/0.54**	**<.001/2.76**	.43/0.31	**<.001/2.20**	.08/0.54
3	Dprime	**<.001/1.33**	.09/0.47	**<.001/1.28**	**.027/0.61**	**.001/0.97**	.70/0.29	**.001/0.83**	.12/0.40
	RT	**<.001/1.94**	>.999/0.07	**<.001//1.53**	.73/0.26	**<.001/1.63**	>.999/0.12	**<.001/1.71**	.72/0.18
4	Dprime	**<.001/2.31**	**<.001/1.08**	**<.001/1.66**	**<.001/0.94**	**.001/1.60**	**.005/0.90**	**<.001/1.88**	.66/0.46
	RT	**<.001/3.30**	**<.001/1.19**	**<.001/2.48**	**<.001/0.86**	**<.001/2.49**	**.04/0.92**	**<.001/2.74**	**.02/1.13**
5	Dprime	**<.001/1.45**	**.006/0.77**	**<.001/1.36**	**.011/0.74**	**.001/0.96**	.07/0.58	**.014/0.74**	.21 0.44
	RT	**<.001/2.14**	.12/0.49	**<.001/1.70**	>.999/0.12	**<.001/2.22**	**.01/0.65**	**<.001/1.82**	.19/0.54

Self-bias (self-related trials against neutral-stranger trials) were obtained in experiments 1–3, and a similar advantage toward the best friend was obtained in experiment 4 and 5. Partner-advantage refers to partner-related trials against neutral-stranger trials. Significant results are shown in bold font

We discovered a surprising partner-advantage and replicated the self-advantage effect. Participants performed better from the beginning block onwards with a partner-associated pairing than with a stranger-associated pairing, and this advantage gradually decreased. However, the self-advantage remained robust through the whole experiment. The partner-advantage in experiment 1 is unexpected because facilitated performance was observed only for familiar people (e.g. friends and mothers) in previous studies (Schäfer et al., 2016; Sui et al., 2012; Sun, Fuentes, Humphreys, & Sui, 2016). Our participants had no social interaction or verbal communication with their assigned partners and never heard each other’s names. In this case, three factors distinguished the roles of partner and neutral stranger: first, the participants briefly met their partners in person; second, the assignment as a partner enjoyed a social labeling that the neutral stranger name did not have; third, the participants co-acted the task with the partner. Which factor was the major contributor to our observed partner-advantage? To answer this question, we isolated these factors in experiments 2 and 3, and tested whether the partner-advantage persisted when the partner did not co-act the task (experiment 2) or even show up (experiment 3).

### Experiment 2

#### Purpose

This experiment was designed to investigate without joint action whether or not a short exposure to a partner was sufficient to induce beneficial partner-related processing.

#### Participants

Another 26 university students participated in this study (13 men and 13 women), all with normal or corrected-to-normal vision. They provided written informed consent and received an explanation of this study after the experiments. In each session there were two participants who had not met each other (i.e. no contact prior to the experiment).

#### Stimuli

The stimuli were identical to those used in experiment 1. The participants confirmed that they did not know either of the other two names before the experiment.

#### Procedure

The procedures were identical to those used in experiment 1 (see Table 1) including the participants being introduced to each other as the co-actors of a task. The only difference was that all the participants performed only 3 blocks individually. We introduced two participants to each other upon their arrival: “This is your partner. You two will do a task individually first, and then perform the task together.” Then one of the participants (A) received instruction about the task and performed the individual condition with an empty chair beside him/her (see Table 1), while the other participant (B) was invited to wait outside the room. Participant A left the room after finishing the task, and then participant B came into the room to receive instruction and to perform the task. Similar to experiment 1, the participants only needed to respond to half of the trials according to the name color. Feedback messages showing “Correct”, “Incorrect”, and “Slow” were displayed in go trials, as in experiment 1. The participants received the feedback “Correct” when they did not respond in no-go trials and “Incorrect” if they pressed any key within 1000 ms. The whole experiment lasted about 30 min.

#### Results

The experiment was identical to experiment 1 except that participants were misled into believing that they were going to perform a task individually and then together with a partner. In reality, they only performed individually. Results obtained from an additional 26 participants were included in the data analysis. Responses faster than 200 ms were excluded, and this eliminated 4.2% of the trials. Only trials with correct responses were included in the analysis of RT, and 12.3% of the trials in which there were erroneous responses were excluded.

**Fig. 3:**
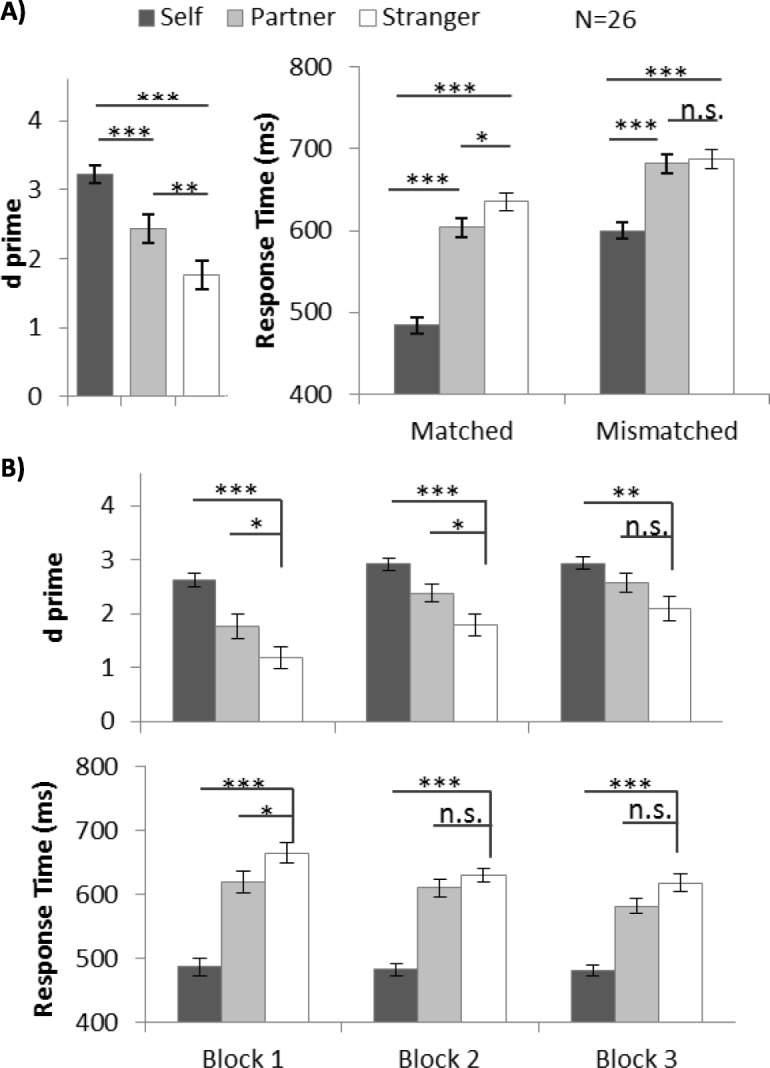
**a** Mean and SE of d prime (*d’*) for different shape categories in experiment 2. **b** Mean and SE of *d’* and response time (RT) (matched trials) for different shape categories for each block in experiment 2 (*p < .05, **p < .01, *** *p* < .001). n.s., not significant

Similar to experiment 1, ANOVA was conducted on *d’* (Fig. 3a) and RT (Fig. 3a) to investigate the identity-associated advantage. Individual participants’ *d’* values are shown in Additional file 7: Figure S7. One-way ANOVA of *d’* showed a significant main effect on shape category (*F* (2, 42) =33.37, *p* < .001, *η*^2^ = 0.57). A post-hoc test showed *d’* was higher for self-related (3.22, *p* < .001) and partner-related stimuli (2.43, *p* = .006) than for stranger-related stimuli (1.77). Average accuracy and accuracy by block are shown for reference in Additional file 2: Figure S2.

Two-way repeated ANOVA for analysis of RT, with factors of shape category and matching judgements, showed a significant interaction effect, *F* (2, 50) = 1438, *p* < .001, *η*^2^ = 0.37. One-way ANOVA was applied separately to matched and mismatched trials and showed a significant main effect of shape category in matched trials, *F* (2, 50) = 110.09, *p* < .001, *η*^2^ = 0.82: RT in self-related trials (484.08 ms) and partner-related trials (603.75 ms) was significantly quicker than in stranger-related trials (635.24 ms, *p* < .001 and *p* = .01 respectively). Self-advantage was observed in mismatched trials (600.16 ms vs 687.40 ms, *p* < .001), but partner-advantage was not (681.88 ms vs 687.40 ms, *p* > .999).

To compare the temporal dynamics of self-advantage and partner-advantage, we examined them block by block by planned pairwise comparison, with Bonferroni adjustments. The results are summarized in Table 2 and Fig. 3b. The self-advantage persisted through 3 blocks: participants responded significantly more accurately and quickly with a self-related stimulus than with stranger-related stimuli. Partner-related associations were significantly stronger than stranger-related associations in block 1 (in *d’* and RT) and block 2 (in *d’*), but not in block 3.

To examine whether self-advantage/partner-advantage was modulated by social context, we compared the performance in experiment 1 (joint condition) and experiment 2 (individual condition). We conducted mixed design ANOVA of *d’* and the RT in matched trials, with the within-subject factor of shape category (self, partner, and stranger) and between-subject factor of social context (joint and individual). The joint condition did not differ from the individual condition for either *d’* (*F* (1, 46) = 3.01, *p* = .09, *η*^2^ = 0.06) or RT (*F* (1, 46) < .001, *p* = .98, *η*^2^ <0.001). And the interaction effect between the two factors was insignificant for both *d’* (*p* = .45) and RT (*p* = .65), indicating similar processing benefits under joint and individual conditions.

Joint action context modulation on identity referential information processing has been largely overlooked in past research. It is surprising that the joint-task condition did not enhance the partner-advantage as social context has been shown to influence cognitive processing as in the joint Simon effect (Dolk et al., 2011; Sebanz, Knoblich, & Prinz, 2003), joint flanker effect (Atmaca, Sebanz, & Knoblich, 2011) and the joint implicit attitude task (Stenzel & Liepelt, 2016). The most relevant finding is the face/agent interference effect identifed by Baess and Prinz (2017), who presented a black or white dot superimposed on a face and required participants to respond to the color of the dot. Faster responses were observed when the background was the participant’s own face, rather than another’s face. Although this own-face advantage was significantly bigger in a joint action setting (when participants responded to one color and the other color was taken care of by a co-actor) than an individual action setting (when participants responded to one color and ignored the other color), the small reaction time difference (i.e. 3 ms) indicated that the effect (if it really exists) is weak. This leads us to speculate that the joint action effect on identity referential information is either small in nature or too weak to be detected with the current paradigm, or is exempted.

Studies have shown that the social states of co-actors modulated self–other integration. A positive relationship enhances the joint Simon effect (Hommel, Colzato, & Van Den Wildenberg, 2009; Müller et al., 2011), and a competitive relationship degrades it (Ruissen & de Bruijn, 2016). Future studies may consider creating cooperation or competition opportunities in addition to sitting participants who are co-performing the task side-by-side to allow a more visible influence from the joint action on the referential processing of different identities.

To summarize experiments 1 and 2, we replicated the partner-advantage effect in different social contexts: partner-association was more accurately and quickly matched than stranger-association. This advantage is the same under individual and joint conditions, which suggests that joint action is not the major contributor. In experiment 2, two factors distinguished the roles of partner and neutral stranger: first, assignment as a partner enjoyed a social label that the neutral stranger’s name did not have; second, participants briefly met their partners in person. Therefore, we tested whether physical presence was crucial in experiment 3 by pairing participants with a partner who never actually appeared.

### Experiment 3

#### Purpose

This experiment aimed to investigate whether identity-advantage toward a stranger introduced as a partner in a joint task persists if the partner is physically absent.

#### Participants

Twenty-two additional students who did not participate in the previous experiments took part in this study (12 men and 10 women), all with normal or corrected-to-normal vision. They provided written informed consent and received an explanation of this study once it had been completed.

#### Stimuli

The stimuli were identical to those used in experiment 2, except that the three names were the participant’s own name, the name of a fake partner, and the neutral stranger name (君明). The fake partners’ names were randomly chosen from experiment 2. Before the experiment the participants confirmed that they knew no one with either of the other two names.

#### Procedure

Participants were misled into believing that they were going to perform a task individually and then together with a partner. They were told to start the individual condition first, because the partner was in the bathroom. In reality, they only performed the individual condition and never met any partner. After the individual condition had been completed, we debriefed the participants about our research purpose. The task procedures were the same as those used for the individual condition in experiment 2. The participants received instructions and practiced 20 trials before 3 blocks of the individual condition. The entire experiment lasted about 30 min.

#### Results

The task procedures were same as those in experiment 2 except that the participants were told to start the individual condition first, because the partner was in the bathroom. In reality, they only performed the task under the individual condition and met no partner.

Results from all 22 new participants were included in the data analysis. Responses faster than 200 ms were excluded, and this eliminated 5.4% of the trials. Only trials with correct responses were included for analysis of RT, and as a result, 6.9% of the trials were excluded because of errors.

**Fig. 4:**
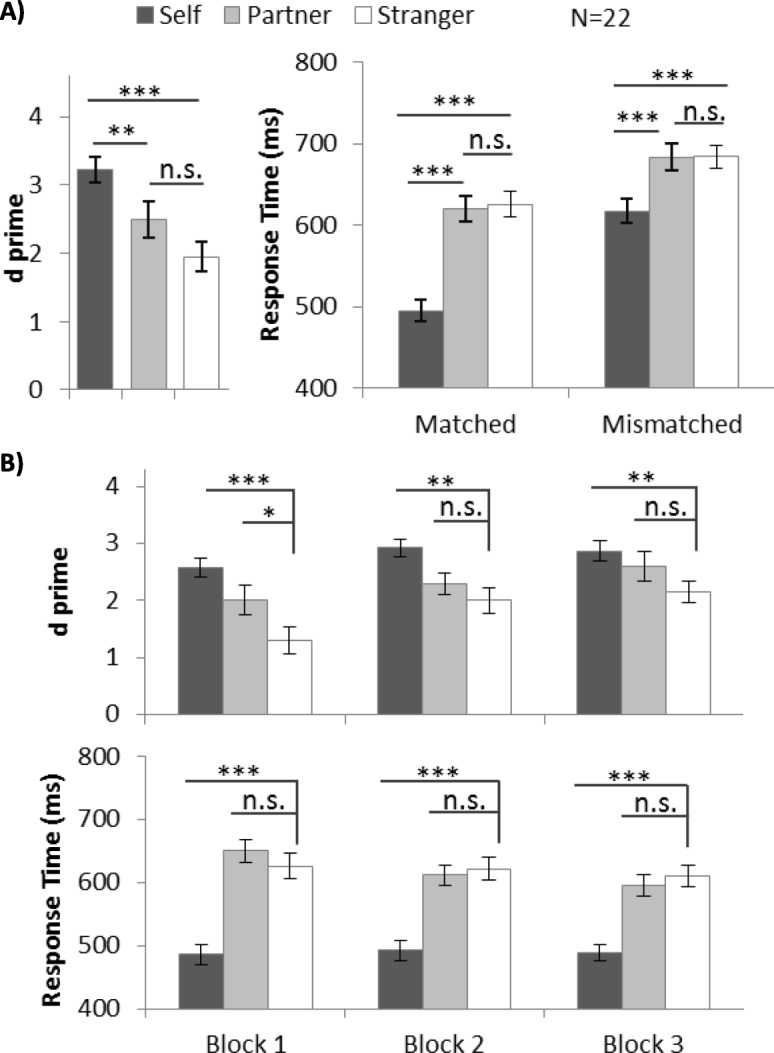
**a** Mean and SE of d prime (*d’*) for different shape categories in experiment 3. **b** Mean and SE of *d’* and response time (RT) (matched trials) for different shape categories for each block in experiment 3 (*p < .05, **p < .01, ***p < .001). n.s., not significant

Figure 4a summarizes the *d’* and RT results obtained in experiment 3. Average accuracy and accuracy by block are shown for reference in Additional file 3: Figure S3. Additional file 8: Figure S8 shows the *d’* values for individual participants. One-way ANOVA of *d’* showed a significant main effect of shape category, *F* (2, 42) = 18.17, *p* < .001, *η*^2^ = 0.46. Post-hoc tests with Bonferroni adjustment showed that the participants performed more accurately in self-related trials (*d’* 3.23) than in stranger-related trials (*d’* 1.95, *p* < .001). However, the *d’* in the partner-related trials (2.49, *p* = .09) did not differ from that in stranger-related trials.

Similarly, the RT results showed a self-advantage but no partner-advantage. We conducted two-way ANOVA with factors of shape category and matching judgements on RT data, and found a significant interaction effect between the two factors (*F* (2, 42) = 12.69, *p* < .001, *η*^2^ = 0.39). Separate one-way ANOVA was conducted on matched and mismatched trials. There was a benefit of self-advantage compared to stranger trials in both matched trials (489.17 ms vs 618.53 ms, *p* < .001) and mismatched trials (617.48 ms vs 683.99 ms, *p* < .001). But the partner-advantage was absent from both matched trials (*p* > .999) and mismatched trials (*p* > .999).

We did a similar block-by-block comparison on *d’* and RT data using a planned pairwise comparison with Bonferroni adjustments, to examine whether partner-advantage was absent throughout the experiment. The results are summarized in Table 2 and Fig. 4b. We observed robust self-advantage through 3 blocks: the participants responded significantly more accurately and quickly to a self-related stimulus than to stranger-related stimuli. There was no difference between partner-related associations and stranger-related associations in any block, except for the brief partner-advantage of *d’* in block 1.

In experiment 3, the assigned partner never appeared, and, on average, there was no difference between the *d’* and response speed in partner-related trials and stranger-related trials. A temporal dynamic analysis revealed a quickly fading initial effect, which was attenuated and disappeared from block 2. The results indicate that the identity of the partner (i.e. social labeling) is insufficient to induce robust prioritized processing, and the physical presence may be a critical precursor in terms of maintaining and stabilizing the partner-advantage.

Experiment 3 provides evidence of the malleability of partner-advantage by eliminating it. This is aligned with past study results showing that self-related priority was more resistant than other-advantage (toward significant others) in manipulations such as reduction in presentation probability (Sui et al., 2014), association with low reward (Sui & Humphreys, 2015), and low-contrast stimulus display (Sui et al., 2012). The current experiment also showed that other-advantage was less robust than self-advantage.

### Experiment 4

#### Purpose

It is unclear how this newly discovered identity-advantage (i.e. partner-advantage) relates to other types of identify referential priority other than self-bias. We repeated experiment 2 except that we replaced “self’s name” with “the best friend’s” name in the hope of better characterizing the relative strength of partner-advantage.

#### Participants

We used data from experiments 1–3 to estimate the sample size required for this control experiment. In experiments 1–3, the effect size (*η*^2^) of the *d’* and response time ranged from 0.45 to 0.84. Because we expected a smaller effect size without self, we used the smallest effect size for sample estimation for experiment 4 (i.e. 0.45). With an estimated *η*^2^ of 0.45, we conducted power analysis using G*Power (Faul et al., 2007), and it yielded the minimum sample size of 9 needed for the *F* test (ANOVA, repeated measures, within factors) for power of 0.8 with the alpha at 0.05. Seventeen additional students who did not participate in previous experiments participated in this study (12 men and 4 women), all with normal or corrected-to-normal vision. They provided written informed consent and received an explanation of this study after it was complete.

#### Stimuli

The stimuli and procedures were identical to experiment 2, except the three names belonging to participant’s best friend’s, a newly met partner (胡弦, i.e. Hu Xian, unisex name), and a neutral stranger (君明, unisex name). The participants confirmed that they knew no one with either one of the other two names before the experiment.

#### Procedure

The participants were misled into believing that they were going to perform a task individually and then together with a partner. In reality, they only performed the task individually. Participants received instructions and practiced 30 trials before they performed 3 blocks individually (150 trials each). The whole experiment lasted about 30 min.

#### Results

Results obtained from 16 of 17 participants were included in the data analysis. One participant was excluded because of her lack of competence at reading Chinese. Responses faster than 200 ms were excluded, eliminating 2.9% of the trials. Only accurate trials were included for analysis of RT, which excluded 6.0% of the total trials.

**Fig. 5:**
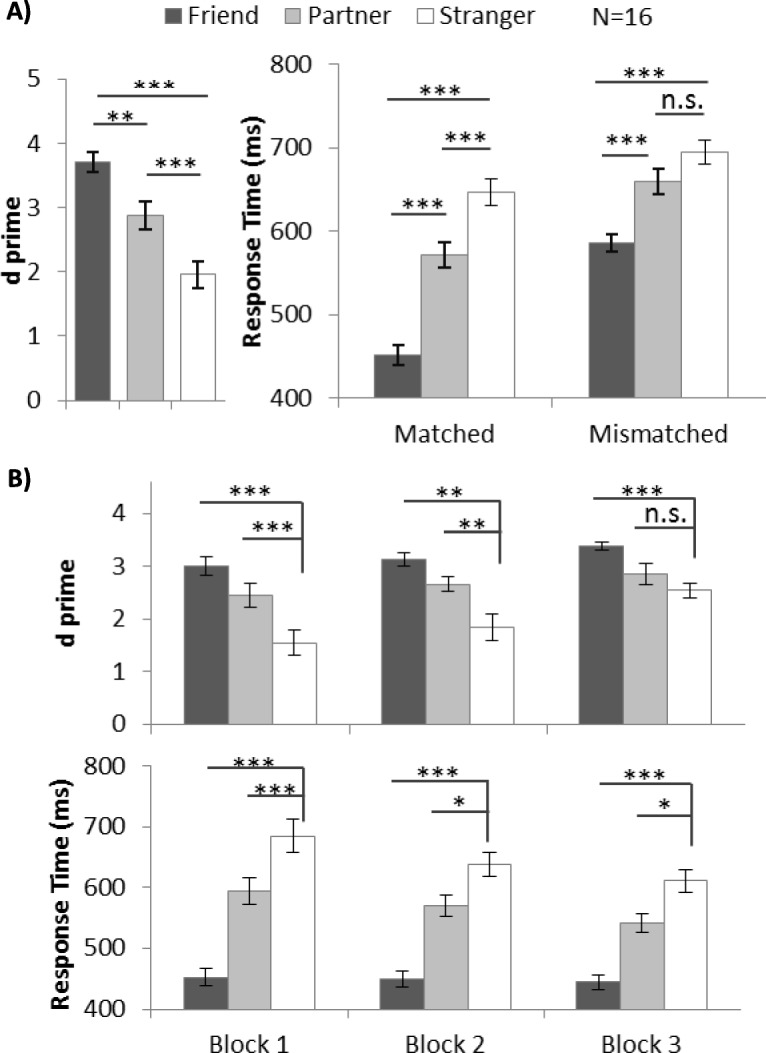
**a** Mean and SE of d prime (*d’*) for different shape categories in experiment 4. **b** Mean and SE of *d’* and response time (RT) (matched trials) for different shape categories for each block in experiment 4. (*p < .05, **p < .01, ***p < .001). n.s., not significant

Figure 5a summarizes the *d’* and RT results obtained in experiment 4. Average accuracy and accuracy by block are shown for reference in Additional file 4: Figure S4. Individual participants’ *d’* values can be found in Additional file 9: Figure S9. One-way ANOVA of *d’* showed a significant main effect of shape category, *F* (2, 30) = 48.96, *p* < .001, *η*^2^ = 0.77. Post-hoc tests with Bonferroni adjustment showed that the participants performed more accurately in trials associated with their best friends (*d’* 3.72) than with strangers (*d’* 1.96, *p* < .001). Similarly, their performance in trials associated with a partner (*d’* 2.88, *p < .001*) was also significantly better than in trials associated with a stranger.

Similarly, the RT results showed an advantage toward the best friend as well as the partner. Two-way repeated ANOVA with within-subject factors of shape category and matching judgements on RT data revealed a significant interaction effect between the two factors (*F* (2, 30) = 9.83, *p* = .001, *η*^2^ = 0.40). One-way ANOVA was conducted separately on matched and mismatched trials. We observed that benefit was endowed more with a best friend than with a stranger in both matched trials (452.01 ms vs 646.78 ms, *p* < .001) and mismatched trials (585.76 ms vs 694.41 ms, *p* < .001). The partner-advantage was observed in matched trials (571.51 ms, *p* > .001), but not in mismatched trials.

To further examine whether this applied to all blocks, planned pairwise tests were conducted and the *d’* and RT results are summarized in Table 2, Fig. 5b. We observed a robust friend-advantage through 3 blocks: the participants responded significantly more accurately and faster to a friend-related stimulus than to stranger-related stimuli. The partner-advantage in terms of *d’* or RT also persisted throughout the entire experiment.

All three identities in experiment 4 belonged to the category “others” (i.e. not self), which enabled us to evaluate the relative strength of the partner-advantage without the influence of “dominant self”. Trials with shapes associated with the newly met partner and with a best friend provided responses that were more accurate and faster than those associated with a stranger (partner-advantage and friend-advantage), and both advantages decreased at the same rate with time. The partner-advantage was significantly weaker than the friend-advantage.

### Experiment 5

#### Purpose

All the participants in experiments 1–4 associated a square with self or best friend, a circle with a partner, and a triangle with a stranger. It is possible that the advantages and disadvantages arise from their associated shapes (e.g. squares are easiest to detect and triangles are the hardest to respond to). In addition, we used a unisex name for the stranger in all the experiments. Ambiguous information on gender might degrade the performance in stranger-associated trials. In experiment 5, we controlled these two possible confounding factors with counterbalanced stimuli. We repeated experiment 4 with two improvements. First, we randomized the shape association with identities for each participant. Second, half of the participants associated a unisex name with a partner and a female name with a stranger, while the other half associated a unisex name with a stranger and a female name with a partner. If the implied gender causes the (dis)advantage, then our counterbalancing across participants will eliminate this unwanted influence. The procedure and task set up were the same as in experiment 4.

#### Participants

We used data from experiment 4 to estimate the sample size required for this control experiment. In experiment 4, where the effect size (*η*^2^) of the *d’* and the RT range were 0.75 and 0.40 respectively, we picked the smallest effect size for sample estimation. Therefore, with an estimated η^2^ of 0.40, we expected an effect size *f* of 0.81. Then a power analysis conducted using G*Power (Faul et al., 2007) yielded the sample size of 10 needed for the *F* test (ANOVA, repeated measures, within factors) for power of 0.8 with the alpha value at 0.05. Due to the need for stimuli counterbalancing (see “Stimuli”), the sample size must be multiples of 12. To have a sample size comparable with those of experiments 1–4, we recruited 24 students (12 men) who did not participate in the previous experiments. They all had normal or corrected-to-normal vision. They provided written informed consent and received an explanation of this study upon completion.

#### Stimuli

The stimuli were identical to those used in experiment 4, except that the shape association with names was randomized and counterbalanced across the participants. There was a total of six sets of possible associations between three shapes (square, circle, and triangle) and three names (best friend, a newly met partner, and a neutral stranger). We also counterbalanced the genders of the names of the partner and the stranger. One unisex name (君明) and one female name (逸晴, i.e. Yi Qing) were used. 君明 was the partner and 逸晴 was the stranger for half of the participants, and the opposite pertained for the other half. With six sets of shape-name combinations and two sets of names, the number of participants needed to be a multiple of 12 (6 × 2). The participants confirmed that they knew no one with either of the other two names before the experiment.

#### Procedure

The procedures were identical to those of experiment 4.

#### Results

The results obtained from 24 participants were all included in the data analysis. Responses faster than 200 ms were excluded, eliminating 2.9% of the trials. Only accurate trials were included for analysis of RT, therefore 7.0% of the trials with incorrect responses were excluded.

**Fig. 6:**
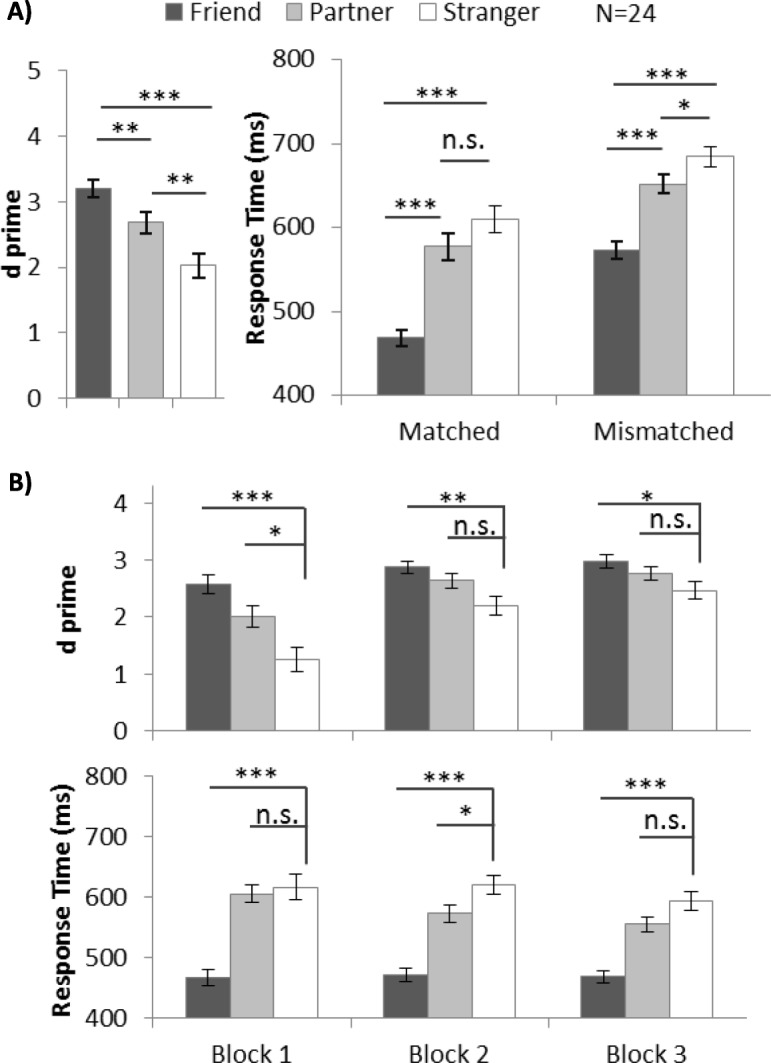
**a** Mean and SE of d prime (*d’*) for different shape categories in experiment 5. **b** Mean and SE of *d’* and response time (RT) (matched trials) for different shape categories for each block in experiment 5 (*p < .05; **p < .01, ***p < .001). All participants’ *d’* values can be found in Additional file 10: Figure S10

The *d’* and response speed results are summarized in Fig. 6a. A similar partner-advantage effect to that observed in experiment 4 was observed in experiment 5. We conducted one-way ANOVA on *d’* and found a significant main effect of shape category, *F* (2, 46) = 25.93, *p* < .001, *η*^2^ = .53. Post-hoc tests with Bonferroni adjustment showed that the participants exhibited greater *d’* in trials associated with their best friends (3.20) than with strangers (2.03, *p* < .001). Similarly, their performance in trials associated with partners (2.68, *p* = .006) was also significantly better than in trials associated with strangers. Average accuracy and accuracy by block are shown for reference in Additional file 5: Figure S5. Individual participants’ *d’* values are shown in Additional file 10: Figure S10.

Two-way repeated ANOVA of the RT data, with within-subject factors of shape category and matching judgements, revealed a significant interaction effect between the two factors (*F* (2, 46) = 32.22, *p* < .001, *η*^2^ = 0.62). One-way ANOVA was conducted separately on matched and mismatched trials. We observed that more benefit was endowed to a best friend than to a stranger in both matched trials (468.56 ms vs 609.45 ms, *p* < .001) and mismatched trials (573.31 ms vs 684.87 ms, *p* < .001). The partner-advantage was observed in the mismatched trials (652.16 ms, *p* = .02), but not in the matched trials (577.22 ms, *p* = .12).

Block-by-block analysis showed that the partner-advantage appeared from the beginning of the experiment and declined gradually. Planned pairwise tests were conducted and the *d’* and RT results are summarized in Table 2, Fig. 6b. We observed a robust friend-advantage through 3 blocks: the participants showed higher *d’* and a faster response with friend-related stimuli than with stranger-related stimuli. The partner-advantage was present in block 1 (in *d’*) and block 2 (in RT), but not in block 3.

In the combined *d’* and RT data, the partner-advantage persisted when name and shape were randomized, indicating that the saliency of a specific stimulus (one particular shape or name) did not contribute to the partner-advantage. As with previous experiments, the strength of the partner-advantage declined and gradually disappeared, suggesting it was less robust relative to the friend-advantage effect.

## Discussion

We investigated identity-associated information processing in different social contexts and provided evidence that identity-priority processing is not limited to self and significant others. The quick establishment of partner-advantage from a brief meeting lasting seconds and without any verbal communication is sufficient to create prioritized processing for a stranger, and its relatively quick reduction in time implies the plasticity of other-advantage. This suggests that identity-associated priority processing is richer than previously thought.

Separate neural pathways have been reported to be involved in in the referential processing of self and others with familiarity and/or social significance as modulating factors. In a functional magnetic resonance imaging (fMRI) study using a similar identity-shape matching task, the ventral medial prefrontal cortex (vmPFC) and the left posterior superior temporal sulcus (STS) were more activated for self-related stimuli, while the dorsal-lateral prefrontal cortex (dlPFC) was more activated for stranger-related stimuli (Sui, Rotshtein, & Humphreys, 2013). Similar results were obtained in various identity-related tasks, such as trait, appearance, and mental state judgements. When we compare self with others (including celebrities and strangers), the vmPFC is more activated for a self-related process and the dorsal mPFC (dmPFC) is more activated for an other-related process (see a meta-analysis and a review article: Denny, Kober, Wager, & Ochsner, 2012; Wagner, Haxby, & Heatherton, 2012). A recent neural stimulation study suggested a causal role of dmPFC in self/other processing: excitatory transcranial direct current stimulation (tDCS) in dmPFC removed the self-advantage over another person (Barack Obama) in a referential memory task (Martin, Dzafic, Ramdave, & Meinzer, 2017). Moreover, familiarity and social significance modulates brain activation levels. In a task where the participants made trait judgements for self, significant others (mother) and familiar celebrities who shared little personal history with the participants (e.g. former U.S. Presidents), the vmPFC activation level recorded by fMRI was highest for self (where the familiarity and social significance with self is at its highest), medium for significant others, and lowest for unfamiliar others (with whom we are least familiar and where the social significance is lowest) (Krienen, Tu, & Buckner, 2010; Moran, Lee, & Gabrieli, 2011). These findings suggest that the other-advantage involves an alternative neural pathway to self-advantage. However, whether these findings apply to a partner without prior familiarity, as in our study, is unknown.

The partner-advantage might be served by a separate mechanism from advantage processing for information associated with self and significant others (i.e. friends, mothers). Several signature observations from our results imply a significant qualitative difference in the effect toward a newly met stranger. First, the brevity of the encounter rules out the involvement of accumulated familiarity, which is a shared feature in advantage processing for self and significant others. This is the first report to show that identity referential salience can be quickly established toward an unfamiliar stranger. Second, the temporal window of processing in our results suggested that the most critical window for initiating a deeper level of processing with a partner in a perceptual matching task was at the beginning. The difference between partner and stranger gradually reduced and became insignificant, while the difference between self/friend and stranger remained robust. We notice that this trend at block 3 mainly came from the performance improvement in stranger trials as the partner and the best friend stimuli are almost responded to at the ceiling level. It is important to be reminded that the identify advantage in the research community is defined as a relative strength between self/significant others/partner and stranger. The establishment of bias can come from the performance increase from self/significant others/partner trials or from the performance reduction in stranger trials or from both. The two sources may be served by different neural mechanisms that are non-separable in current study. It will be an interesting direction to experimentally disentangle these two sources in future studies. In other words, the advantage quickly decreased over time and did not survive for long in the same way as the self/friend-advantage. This implied that the advantage might depend on short-lived neural circuits designed to respond to a new social situation. In addition, prolonged exposure or an opportunity to co-act in a joint task did not further strengthen the effect, which is opposite to what one would expect from the learning effect. To sum up, it is plausible to assume that these distinctions require a different component to account for the relationship involved in a longer personal history (e.g. self and significant others).

What might be a possible platform for such quickly established selectivity? It is well-known that a first impression can be formed within one second (Willis & Todorov, 2006). A short exposure to a face for less than one minute is sufficient to make an accurate judgment of various features including attractiveness (Willis & Todorov, 2006), health (Rule, Garrett, & Ambady, 2010), personality traits (Naylor, 2007; Olivola & Todorov, 2010), intelligence (Carney, Colvin, & Hall, 2007), competence (Naylor, 2007), sexual orientation (Rule & Ambady, 2008; Rule, Ambady, & Hallett, 2009), and religious group membership (Rule et al., 2010). In the current study (experiments 1, 2, 4, and 5), the introduction is long enough to allow participants to create an impression from the partners’ appearance and nonverbal cues (eye contact, facial expression, and body language).

The amygdala and the posterior cingulate cortex (PCC) were previously identified as crucial for forming first impressions from faces and verbal descriptions (Schiller, Freeman, Mitchell, Uleman, & Phelps, 2009). In particular, the amygdala is associated with the processing of nonverbal personal information such as facial and body movements; while the PCC responds to verbal information such as descriptions of one’s social behaviors (Kuzmanovic et al., 2012). In addition to being an important contributor to the other-referential advantage, the dmPFC is also involved in impression formation. A transcranial magnetic stimulation (TMS) study (Ferrari et al., 2014) showed that the dmPFC played a crucial role in integrating impressions obtained from nonverbal and verbal stimuli. The participants first used verbal descriptions to form positive or negative impressions of faces, and they judged whether a new adjective fitted the impressions. Responses slowed when participants received TMS over the dmPFC, compared with TMS over the inferior frontal gyrus or over a control site (vertex). Interestingly, no impairment effect from TMS was observed when impressions were formed from face or verbal description alone. This study indicated that the mechanisms of impression formation for facial and verbal information were disassociated. The processing advantage in the present study was established between participants who met briefly without any verbal communication. More effort is needed to explore whether the partner-advantage has a neural network that overlaps with the impression formation of nonverbal stimuli.

From the depth-of-processing perspective (Baddeley & Woodhead, 1982; Craik & Tulving, 1975), we speculate that the additional information obtained from first impressions can induce partner name processing at a deeper level than stranger names. It is reported that facial memory can be enhanced by deeper levels of processing, for example in visual imagery (Swann & Miller, 1982), providing contextual and personality information (Baddeley & Woodhead, 1982), and trait and facial physical feature judgments (Parkin & Hayward, 1983). Deeper processing also promotes identity referential information coding such as evidence showing that name-face association is improved by deeper semantic processing (Troyer, Häfliger, Cadieux, & Craik, 2006), visual imagery, and affective judgments (Yesavage, Rose, & Bower, 1983). Face-related processing is involved in these previous results, which provided a possible candidate to illustrate why a brief physical presence is sufficient to evoke deeper processing in name-shape association in the current study.

Other possible candidates for generating the partner-advantage effect may include memory structure, social facilitation, peer pressure, or social bonding. It has been suggested that self-related memory includes contributions from autobiographical knowledge and hierarchical structure, and the frequent use of self-related information generates quick access to memory structures and efficient coding (Conway & Pleydell-Pearce, 2000; Symons & Johnson, 1997). Similar benefits may be exerted on the partnership relationship and future investigations are needed to elucidate this possibility. Cognitive performance, as in the Stroop task, is improved with the presence of an audience, even when the audience is invisible (Huguet, Galvaing, Monteil, & Dumas, 1999). Similarly, a participant sitting next to another person performing a similar task can have a beneficial effect on performance compared with a person acting alone (Böckler, Knoblich, and Sebanz, 2012), possibly through shared attention and social bonding (Wolf, Launay, and Dunbar, 2016). Identity-related bias in shape-matching tasks has long been assumed to occur during perceptual selection (Sui & Humphreys, 2015; Sui & Humphreys, 2015) until a recent study demonstrated that there is also a decision-level origin (Macrae, Visokomogilski, Golubickis, Cunningham, and Sahraie, 2017). Social facilitation might be a constituent part of this process, but it is unlikely to be the main modulator because social presence should enhance the overall task performance, which includes all identities. There has been no report on selective facilitation caused by identity to the best of our knowledge, and it will require additional investigations to clarify the role from social facilitation.

In our experiments, we controlled the frequency effect by ensuring that our name choices of “partner” and “stranger” were novel to all the participants (i.e. they did not know anyone with this name). We also used two-character-names to control word length in the current study. Similarly, we drew the conclusion that the processing advantage we observed was not induced by the physical attributions of identity-associated labels.

The current study suggests directions for a further exploration of beneficial processing for non-self-identities. To test whether other-advantage is modulated by the depth of processing, future manipulations of partner-related identity can include information from shallow level processing such as profile photos to deep level processing such as personality trait judgment. Another intriguing direction would be to distinguish and associate two contributors for referential processing: social significance and familiarity. Our result from named-partner trials suggested that its benefit over a stranger-trial decays quickly. This implies that the familiar identity advantage must have been established via a different route. The interplay between social significance and familiarity warrants a closer examination of how they independently and interactively modulate the identity-related advantage.

## Additional files

Additional file 1: **Figure S1.** Accuracy results in experiment 1. (A) Mean and SE of accuracy for different shape categories in experiment 1. (B) Mean and SE of accuracy (matched trials) for different shape categories for each block in experiment 1 (*p < .05, **p < .01, ***p < .001). (PPTX 77 kb)

Additional file 2: **Figure S2.** Accuracy results in experiment 2. (A) Mean and SE of accuracy for different shape categories in experiment 2. (B) Mean and SE of accuracy (matched trials) for different shape categories for each block in experiment 2. (*p < .05, **p < .01, ***p < .001). (PPTX 77 kb)

Additional file 3: **Figure S3.** Accuracy results in experiment 3. (A) Mean and SE of accuracy for different shape categories in experiment 3. (B) Mean and SE of accuracy (matched trials) for different shape categories for each block in experiment 3. (*p < .05, *p* < .01, ***p < .001). (PPTX 66 kb)

Additional file 4: **Figure S4.** Accuracy results in experiment 4. (A) Mean and SE of accuracy for different shape categories in experiment 4. B) Mean and SE of accuracy (matched trials) for different shape categories for each block in experiment 4. (*p < .05, *p* < .01, ***p < .001). (PPTX 78 kb)

Additional file 5: **Figure S5.** Accuracy results in experiment 5. (A) Mean and SE of accuracy for different shape categories in experiment 5. (B) Mean and SE of accuracy (matched trials) for different shape categories for each block in experiment 5. (*p < .05, *p* < .01, ***p < .001). (PPTX 83 kb)

Additional file 6: **Figure S6.** The *d’* value of different shape categories for each participant in experiment 1. From left to right, participants were arranged from those who showed strong self-advantage and partner-advantage to those who showed a weaker effect. (PPTX 93 kb)

Additional file 7: **Figure S7.** The *d’* value of different shape categories for each participant in experiment 2. From left to right, participants were arranged from those who showed strong self-advantage and partner-advantage to those who showed a weaker effect. (PPTX 85 kb)

Additional file 8: **Figure S8.** The *d’* value of different shape categories for each participant in experiment 3. From left to right, participants were arranged from those who showed strong self-advantage and partner-advantage to those who showed a weaker effect. (PPTX 85 kb)

Additional file 9: **Figure S9.** The *d’* value of different shape categories for each participant in experiment 4. From left to right, participants were arranged from those who showed strong friend-advantage and partner-advantage to those who showed a weaker effect. (PPTX 81 kb)

Additional file 10: **Figure S10.** The *d’* value of different shape categories for each participant in experiment 5. From left to right, participants were arranged from those who showed strong friend-advantage and partner-advantage to those who showed a weaker effect. (PPTX 84 kb)

## Data Availability

Individual participants’ results are included in the Additional Files. Data and stimuli materials are available upon request.
