# Supplementary figures and images for: Saliency at first sight: instant identity referential advantage toward a newly met partner
Source: Cogn Res Princ Implic. 2019 Nov 4;4:42. doi: 10.1186/s41235-019-0186-z (PMC6828888; doi:10.1186/s41235-019-0186-z)

## Slide 1
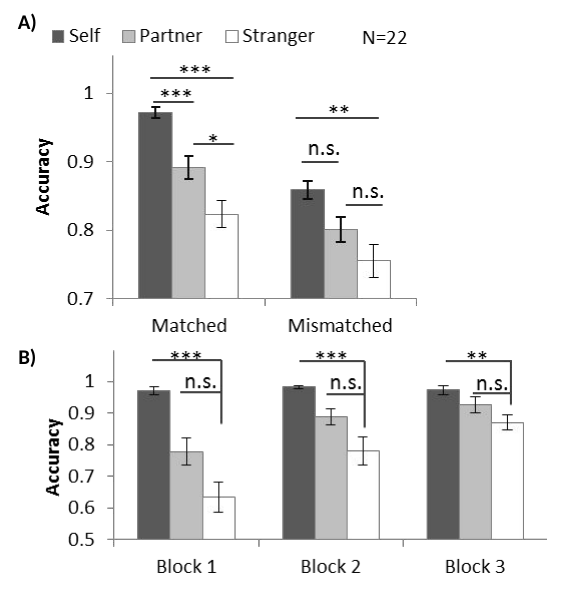

A)
B)

Supplement: Supplementary file 1 — Figure S1. Accuracy results in experiment 1. (A) Mean and SE of accuracy for different shape categories in experiment 1. (B) Mean and SE of accuracy (matched trials) for different shape categories for each block in experiment 1 (*p < .05, **p < .01, ***p < .001). (PPTX 77 kb) [file 41235_2019_186_MOESM1_ESM.pptx]

## Slide 1
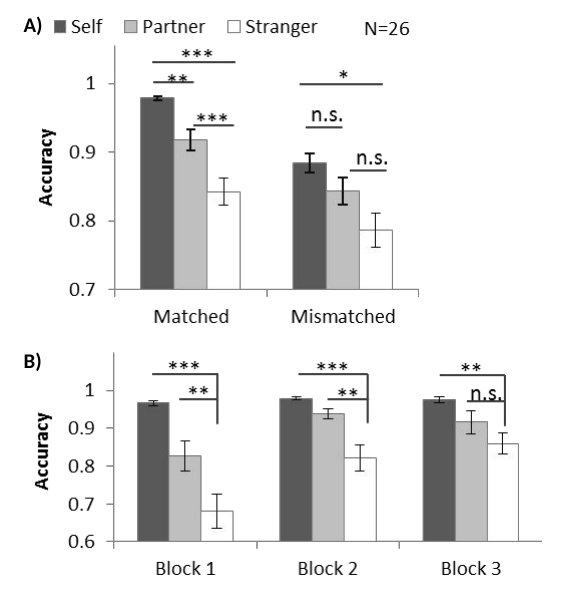

A)
B)

Supplement: Supplementary file 2 — Figure S2. Accuracy results in experiment 2. (A) Mean and SE of accuracy for different shape categories in experiment 2. (B) Mean and SE of accuracy (matched trials) for different shape categories for each block in experiment 2. (*p < .05, **p < .01, ***p < .001). (PPTX 77 kb) [file 41235_2019_186_MOESM2_ESM.pptx]

## Slide 1
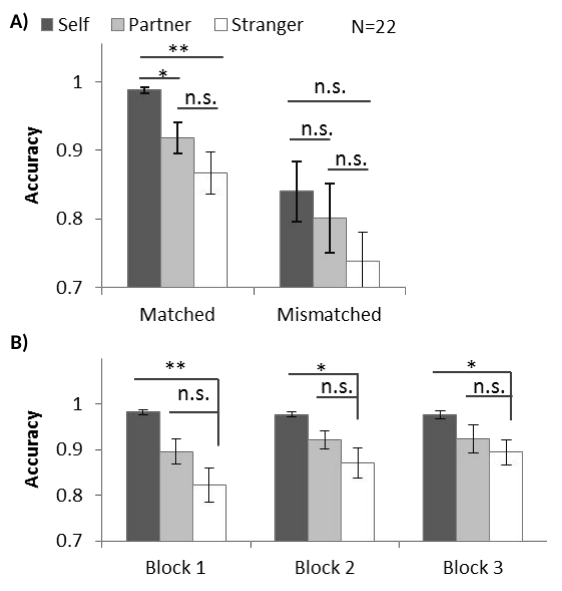

A)
B)

Supplement: Supplementary file 3 — Figure S3. Accuracy results in experiment 3. (A) Mean and SE of accuracy for different shape categories in experiment 3. (B) Mean and SE of accuracy (matched trials) for different shape categories for each block in experiment 3. (*p < .05, p < .01, ***p < .001). (PPTX 66 kb) [file 41235_2019_186_MOESM3_ESM.pptx]

## Slide 1
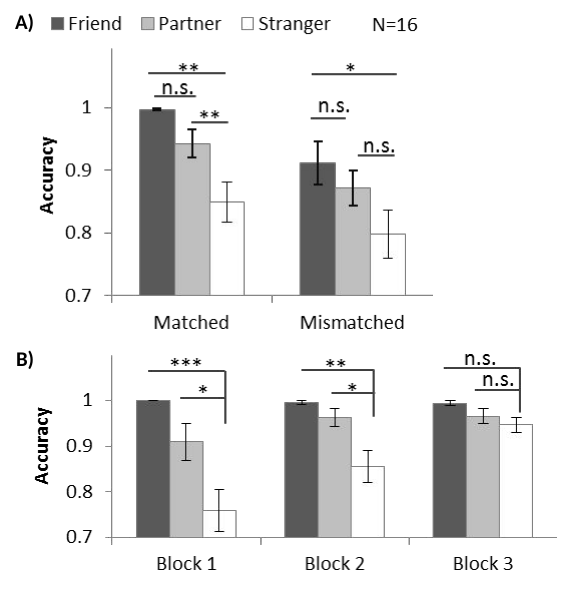

A)
B)

Supplement: Supplementary file 4 — Figure S4. Accuracy results in experiment 4. (A) Mean and SE of accuracy for different shape categories in experiment 4. B) Mean and SE of accuracy (matched trials) for different shape categories for each block in experiment 4. (*p < .05, p < .01, ***p < .001). (PPTX 78 kb) [file 41235_2019_186_MOESM4_ESM.pptx]

## Slide 1
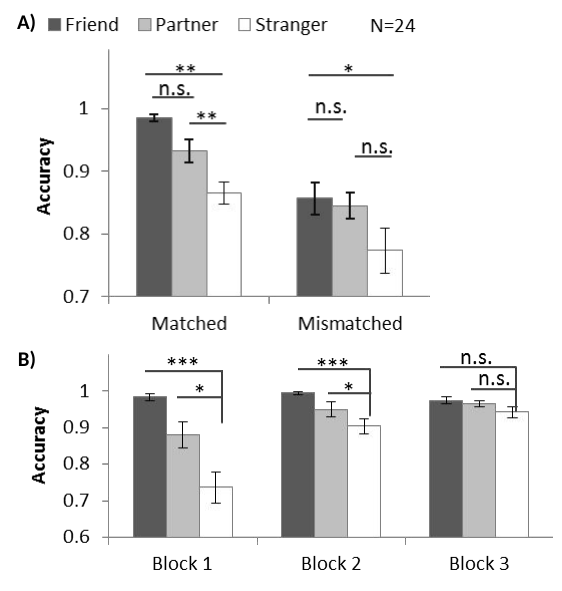

A)
B)

Supplement: Supplementary file 5 — Figure S5. Accuracy results in experiment 5. (A) Mean and SE of accuracy for different shape categories in experiment 5. (B) Mean and SE of accuracy (matched trials) for different shape categories for each block in experiment 5. (*p < .05, p < .01, ***p < .001). (PPTX 83 kb) [file 41235_2019_186_MOESM5_ESM.pptx]

## Slide 1
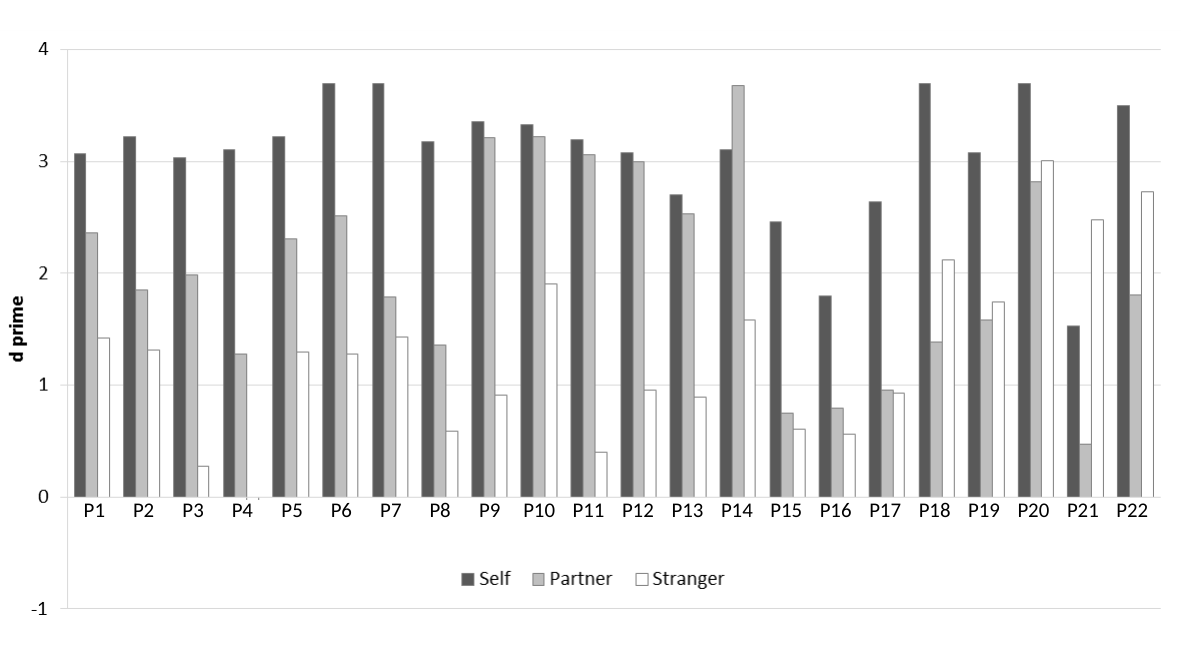

| P1 | P2 | P3 | P4 | P5 | P6 | P7 | P8 | P9 | P10 | P11 | P12 | P13 | P14 | P15 | P16 | P17 | P18 | P19 | P20 | P21 | P22 |
| --- | --- | --- | --- | --- | --- | --- | --- | --- | --- | --- | --- | --- | --- | --- | --- | --- | --- | --- | --- | --- | --- |

Supplement: Supplementary file 6 — Figure S6. The d’ value of different shape categories for each participant in experiment 1. From left to right, participants were arranged from those who showed strong self-advantage and partner-advantage to those who showed a weaker effect. (PPTX 93 kb) [file 41235_2019_186_MOESM6_ESM.pptx]

## Slide 1
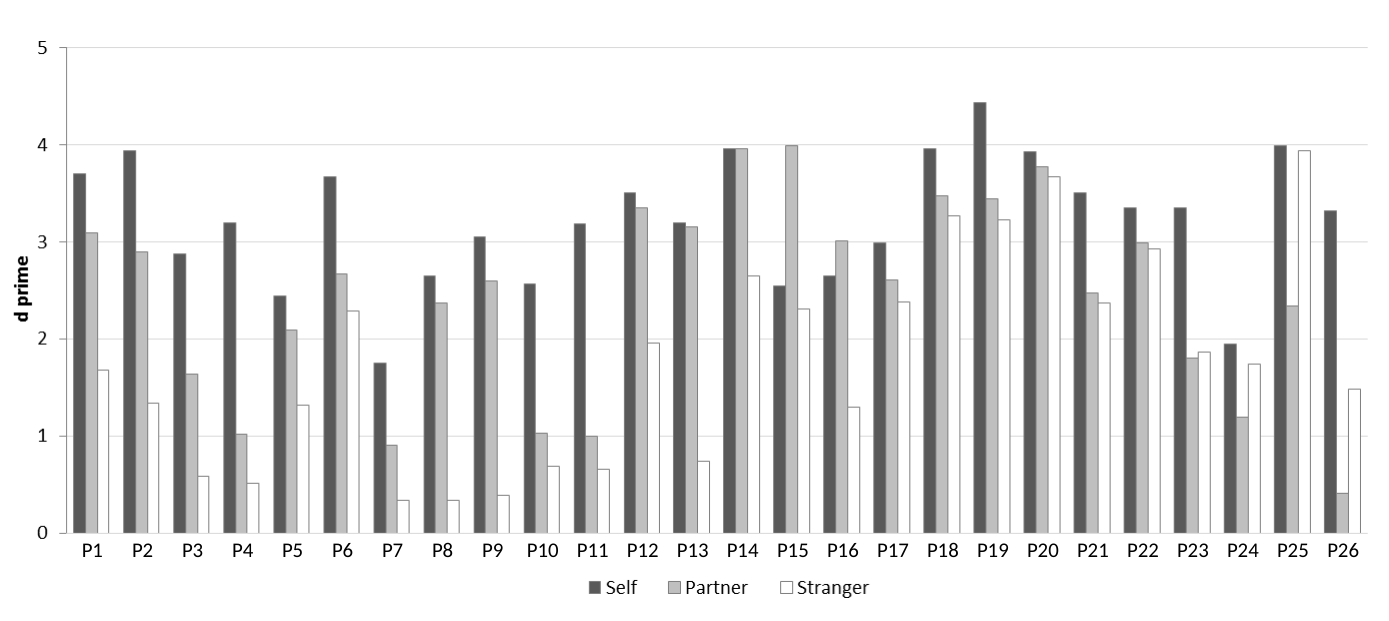

| P1 | P2 | P3 | P4 | P5 | P6 | P7 | P8 | P9 | P10 | P11 | P12 | P13 | P14 | P15 | P16 | P17 | P18 | P19 | P20 | P21 | P22 | P23 | P24 | P25 | P26 |
| --- | --- | --- | --- | --- | --- | --- | --- | --- | --- | --- | --- | --- | --- | --- | --- | --- | --- | --- | --- | --- | --- | --- | --- | --- | --- |

Supplement: Supplementary file 7 — Figure S7. The d’ value of different shape categories for each participant in experiment 2. From left to right, participants were arranged from those who showed strong self-advantage and partner-advantage to those who showed a weaker effect. (PPTX 85 kb) [file 41235_2019_186_MOESM7_ESM.pptx]

## Slide 1
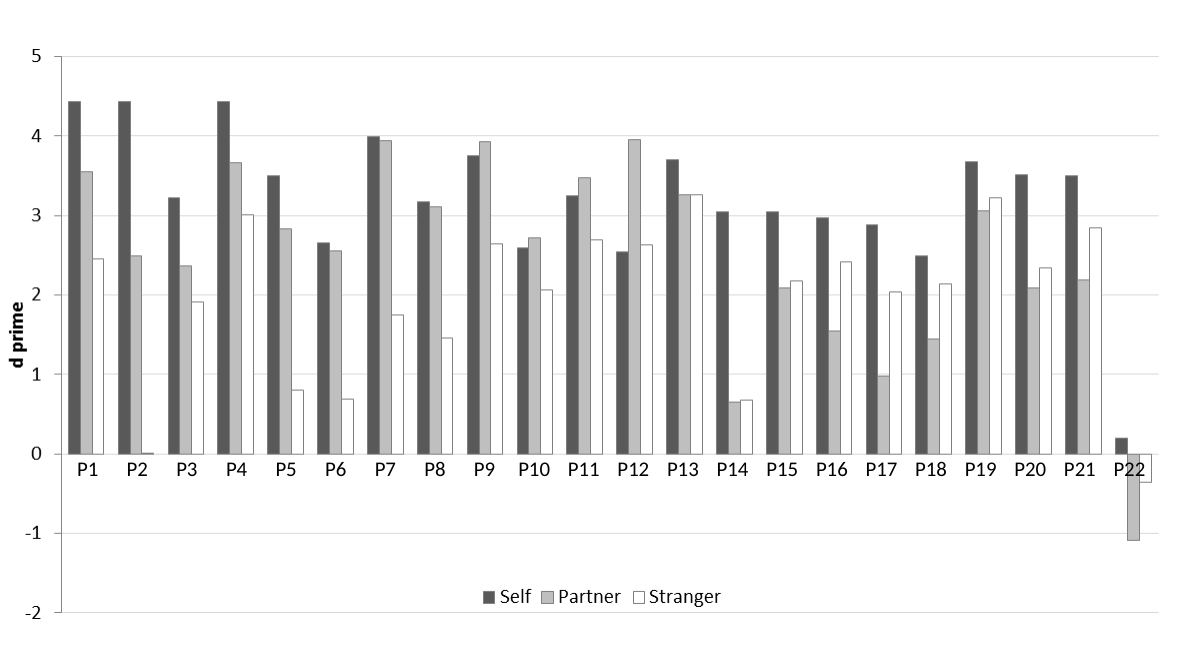

| P1 | P2 | P3 | P4 | P5 | P6 | P7 | P8 | P9 | P10 | P11 | P12 | P13 | P14 | P15 | P16 | P17 | P18 | P19 | P20 | P21 | P22 |
| --- | --- | --- | --- | --- | --- | --- | --- | --- | --- | --- | --- | --- | --- | --- | --- | --- | --- | --- | --- | --- | --- |

Supplement: Supplementary file 8 — Figure S8. The d’ value of different shape categories for each participant in experiment 3. From left to right, participants were arranged from those who showed strong self-advantage and partner-advantage to those who showed a weaker effect. (PPTX 85 kb) [file 41235_2019_186_MOESM8_ESM.pptx]

## Slide 1
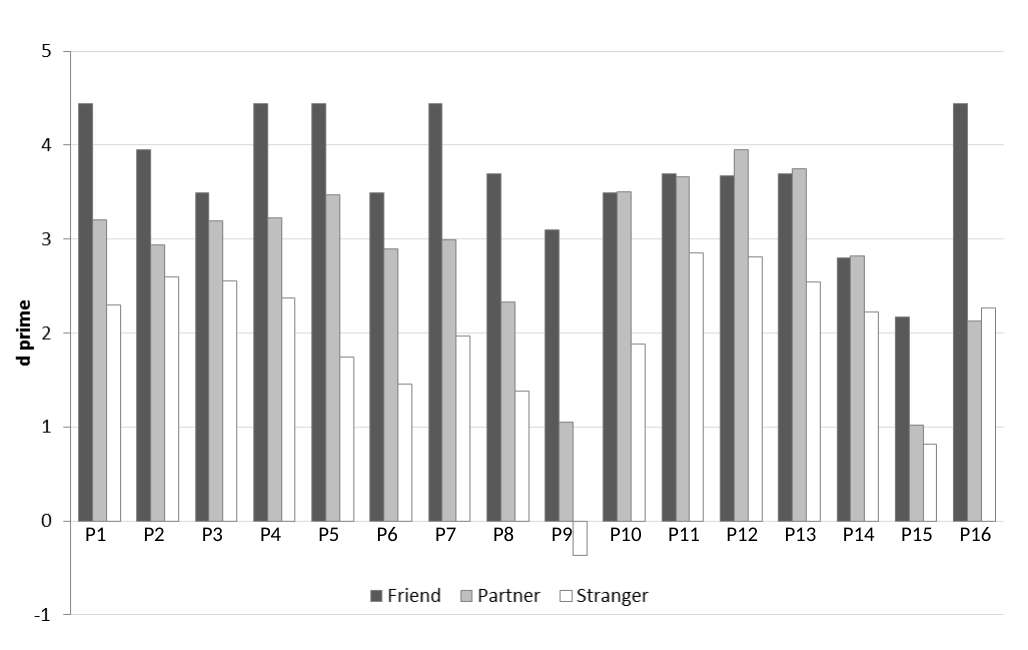

| P1 | P2 | P3 | P4 | P5 | P6 | P7 | P8 | P9 | P10 | P11 | P12 | P13 | P14 | P15 | P16 |
| --- | --- | --- | --- | --- | --- | --- | --- | --- | --- | --- | --- | --- | --- | --- | --- |

Supplement: Supplementary file 9 — Figure S9. The d’ value of different shape categories for each participant in experiment 4. From left to right, participants were arranged from those who showed strong friend-advantage and partner-advantage to those who showed a weaker effect. (PPTX 81 kb) [file 41235_2019_186_MOESM9_ESM.pptx]

## Slide 1
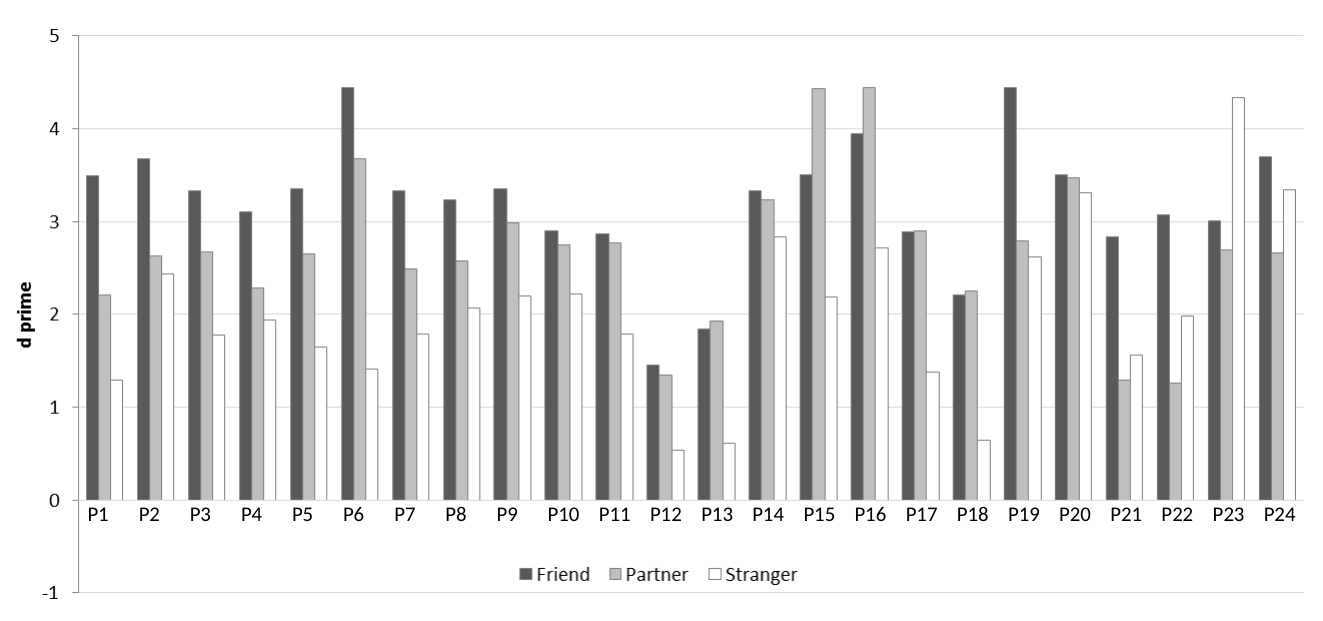

| P1 | P2 | P3 | P4 | P5 | P6 | P7 | P8 | P9 | P10 | P11 | P12 | P13 | P14 | P15 | P16 | P17 | P18 | P19 | P20 | P21 | P22 | P23 | P24 |
| --- | --- | --- | --- | --- | --- | --- | --- | --- | --- | --- | --- | --- | --- | --- | --- | --- | --- | --- | --- | --- | --- | --- | --- |

Supplement: Supplementary file 10 — Figure S10. The d’ value of different shape categories for each participant in experiment 5. From left to right, participants were arranged from those who showed strong friend-advantage and partner-advantage to those who showed a weaker effect. (PPTX 84 kb) [file 41235_2019_186_MOESM10_ESM.pptx]
